# Case report: Primary pericardial angiosarcoma, a rare cause of cardiac tamponade

**DOI:** 10.3389/fcvm.2024.1344975

**Published:** 2024-02-13

**Authors:** Ling-Yun Kong, Xiao-Zheng Cui, Wei Xiang, Xiu-Juan Wang, Fang Liu

**Affiliations:** Cardiovascular Center, School of Clinical Medicine, Beijing Tsinghua Changgung Hospital, Tsinghua University, Beijing, China

**Keywords:** pericardial neoplasm, pericardial angiosarcoma, cardiac tamponade, echocardiography, outcome

## Abstract

Primary pericardial angiosarcoma is a rare malignancy of the pericardium with variable clinical features and imaging characteristics. Herein, we report a case of histopathologically confirmed pericardial angiosarcoma in a 66-year-old man. The patient developed cardiac tamponade in a short time period. The transthoracic echocardiography showed the presence of multiple irregular echodensities, heterogeneous in echogenicity, encasing the apex of both ventricles in the pericardial space, initially misinterpreted as pericardial effusion. The patient died of cardiogenic shock despite undergoing a surgical pericardiectomy. Pericardial angiosarcoma can manifest as a mass obliterating the pericardial sac, rather than the typical pericardial effusion observed on echocardiography. Multimodality imaging studies aid in diagnosing primary pericardial angiosarcoma, but the final diagnosis relies on tissue histopathology.

## Introduction

Primary pericardial angiosarcoma is a rare malignancy of the pericardium. Its clinical features and imaging characteristics are variable. We report a case of an adult male who developed primary pericardial angiosarcoma, diagnosed 3 years after resection of colon carcinoma, and describe the multimodality imaging findings including unusual characteristics on echocardiography.

## Case presentation

**Figure 1:**
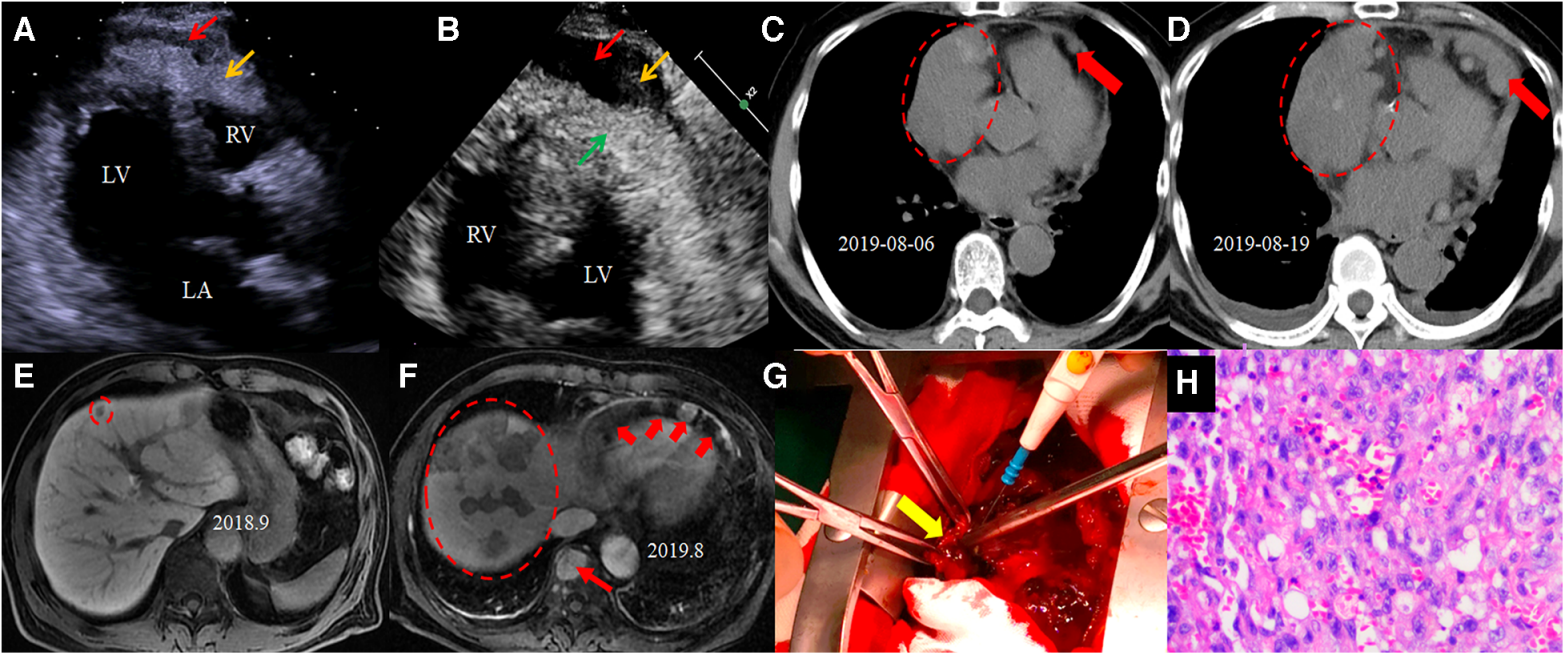
Iconography of the patient. The echocardiogram shows an echolucency (red arrow) and heterogeneous echodensities (yellow arrow) in the pericardial space in parasternal long axis view (**A**) and in apical four-chamber view (**B**) Chest CT (**C**) before admission reveals a heterogeneous mass (dotted ellipse) and pericardial nodules (red arrow). The repeat CT after admission (**D**) reveals an enlarged pericardial mass (dotted ellipse) and nodules (red arrow). The abdominal magnetic resonance hepatobiliary phase 1 year ago shows a low-signal nodule in the liver (dotted ellipse) on T2 weighted image. The magnetic resonance after this admission (**F**) shows multiple lesions involving the liver (dotted ellipse), spine (thin arrow), and pericardium (thick arrow). The gross specimen (**G**) shows a haemorrhagic solid tumour (yellow arrow) infiltrating the ventricular myocardium, and only partial pericardiectomy was performed. Histopathology (**H**) examinations confirmed the pericardial tissue to be angiosarcoma.

A 69-year-old male exhibited symptoms of fever, dry cough, and dyspnoea for a duration of 3 weeks. His past medical history included an uneventful surgery for a well-differentiated carcinoma of the colon (T1bN0M0, stage IA) 3 years ago, as well as hypertension and hyperlipidaemia. He quit cigarette smoking 20 years ago. No particular social history or family history of cancer was reported. A physical examination at admission revealed a body temperature of 37.6°C, a pulse rate of 109 beats per minute, a respiratory rate of 25 breaths per minute, and a blood pressure of 108/75 mmHg. The physical exam was otherwise unremarkable, except for tachycardia and muffled heart sounds. The laboratory findings showed leucocytosis (9.94 × 10^9^/L) with neutrophilia (77.8%), an elevated C-reactive protein level (108.12 mg/L), and an elevated erythrocyte sedimentation rate (89 mm/h). The level of hypersensitivity cardiac troponin-T was mildly elevated at 0.04 ng/ml (reference range 0–0.024 ng/ml). The electrocardiogram showed sinus tachycardia. Transthoracic echocardiography demonstrated a normal left ventricular ejection fraction (63%) and bi-atrial enlargement. Heterogeneous echodensities in the apical pericardial space were noted (Figures 1A,B). Chest computed tomography (CT) after admission revealed newly developed pulmonary and hepatic lesions compared with the CT findings 1 year prior, when he had regular post-operation follow-up for colon cancer. The CT scan also revealed a heterogeneous mass located on the right side, lateral to the pericardium, which is larger than that observed 2 weeks prior on the outpatient CT scan (Figures 1C,D). Enhanced abdominal magnetic resonance imaging revealed multiple lesions in the liver and vertebral bodies (Figures 1E,F), which also included part of the heart and revealed ill-defined solid lesions involving the pericardial sac. The patient developed worsening dyspnoea, hypotension, and tachycardia and subsequently underwent a surgical pericardiectomy 25 days after admission. The pericardial space was found to be severely constricted by the haemorrhagic tumour tissues, but no pericardial effusion was present (Figure 1G). A palliative partial pericardiectomy was performed as the tumour had already infiltrated the myocardium and was unresectable. The diagnosis of pericardial angiosarcoma was confirmed through post-operative pathology of the pericardial tissue (Figure 1H), characterized by vimentin (+), AE1/AE3 (−), CD31 (+), CD34 (+), EMA (−), D2-40 (partially+), calretinin (−), and Ki-67 (25%+) on immunohistochemistry. Unfortunately, the patient died on the 12th day after the surgery due to cardiogenic shock and cardiac arrest. Table 1 lists the timeline and clinical course of the patient.

**Table 1:** Timeline of the patient.

Timepoint	Event
3 years ago	The patient received an uneventful surgery for a well-differentiated carcinoma of the colon (T1bN0M0, stage IA).
1 year ago	The patient was asymptomatic and underwent chest CT for colon cancer surveillance.
3 weeks ago	The patient developed recurrent fever, cough and dyspnea.
Day 1 (hospital presentation)	The patient was admitted for fever, cough, and dyspnoea. Empirical treatment with antibiotics was ineffective. He underwent echocardiography, chest computed tomography, and enhanced abdominal magnetic resonance imaging examinations.
Day 25	The patient underwent partial pericardiectomy as the tumor was inseparable from the myocardium, and no pericardial effusion was found.
Day 37	The patient died of cardiogenic shock.

## Discussion

Angiosarcomas are the most commonly reported primary malignant cardiac tumours, but primary pericardial angiosarcomas are extremely rare (1). Pericardial angiosarcoma has an insidious but aggressive nature. It is often diagnosed at a late stage. In our case, the pericardial angiosarcoma was diagnosed by surgical histopathology. Due to the patient's history of colon cancer and newly diagnosed metastatic lesions involving the liver and vertebrae, metastatic pericardial malignancy from colon cancer was at the top of our differential diagnoses. However, the pathological analysis showed no evidence of colon cancer metastasis but primary angiosarcoma involving the pericardium. The metastatic lesions in the liver and the vertebrae could possibly come from the pericardial angiosarcoma, although no autopsy report is available to confirm this.

Pericardial angiosarcomas often manifest as pericardial effusions rather than a visible mass, posing a diagnostic challenge due to the possibility of other cardiac abnormalities also causing pericardial effusions (2, 3). Pericardial mesothelioma can also masquerade as pericardial effusion (3). Echocardiography is a readily available imaging modality for evaluating pericardial effusion (4). In our case, the primary pericardial angiosarcoma was echocardiographically characterized by pericardial effusion mixed with heterogeneous echodensities due to a tumour encasing the heart. The tumour growth into the pericardial space may be misinterpreted as a pericardial fat pad, which is a benign condition. Multimodality imaging is warranted (5, 6).

## Conclusions

Pericardial angiosarcoma is a rare malignant pericardial neoplasm. It may manifest as a mass obliterating the pericardial sac, rather than pericardial effusion as usually seen on echocardiography. Multimodality imaging studies can assist in the diagnosis of primary pericardial angiosarcoma, but the final diagnosis relies on tissue histopathology.

## Data Availability

The raw data supporting the conclusions of this article will be made available by the authors, without undue reservation.

## Supplementary material

Supplementary Video S1 The apical four-chamber view shows heterogeneous echodensities in the pericardial space.

Supplementary Video S2 The apical two-chamber view shows echolucency and heterogeneous echodensities in the pericardial space resembling effusion.

## References

[1] Guo Y Liu Q Wu H. Primary cardiac tumor: a case report of right atrial angiosarcoma and review of the literature. Front Oncol. (2023) 13:1164153. 10.3389/fonc.2023.1164153 37305576 PMC10250602

[2] Schusler R Meyerson SL. Pericardial disease associated with malignancy. Curr Cardiol Rep. (2018) 20(10):92. 10.1007/s11886-018-1040-5 30128844

[3] Kong L Li Z Wang J Lv X. Echocardiographic characteristics of primary malignant pericardial mesothelioma and outcomes analysis: a retrospective study. Cardiovasc Ultrasound. (2018) 16(1):7. 10.1186/s12947-018-0125-z 29695235 PMC5922299

[4] Adler Y Charron P Imazio M Badano L Barón-Esquivias G Bogaert J 2015 ESC guidelines for the diagnosis and management of pericardial diseases: the task force for the diagnosis and management of pericardial diseases of the European Society of Cardiology (ESC) endorsed by the European Association for Cardio-Thoracic Surgery (EACTS). Eur Heart J. (2015) 36(42):2921–64. 10.1093/eurheartj/ehv318 26320112 PMC7539677

[5] Zhao Y Tian F Ge Z Pan C Shu X. Multimodality imaging for the diagnosis of primary pericardial angiosarcoma. Circ J. (2023) 87(9):1250. 10.1253/circj.CJ-23-0397 37482410

[6] Senthil Kumaran S Asif AA Hussain H Chatterjee T. Pericardial angiosarcoma: a diagnostic challenge. Cureus. (2021) 13(5):e15350. 10.7759/cureus.15350 34235027 PMC8244580

