# Supplementary figures and images for: Case report: Primary pericardial angiosarcoma, a rare cause of cardiac tamponade
Source: Front Cardiovasc Med. 2024 Feb 13;11:1344975. doi: 10.3389/fcvm.2024.1344975 (PMC10897014; doi:10.3389/fcvm.2024.1344975)

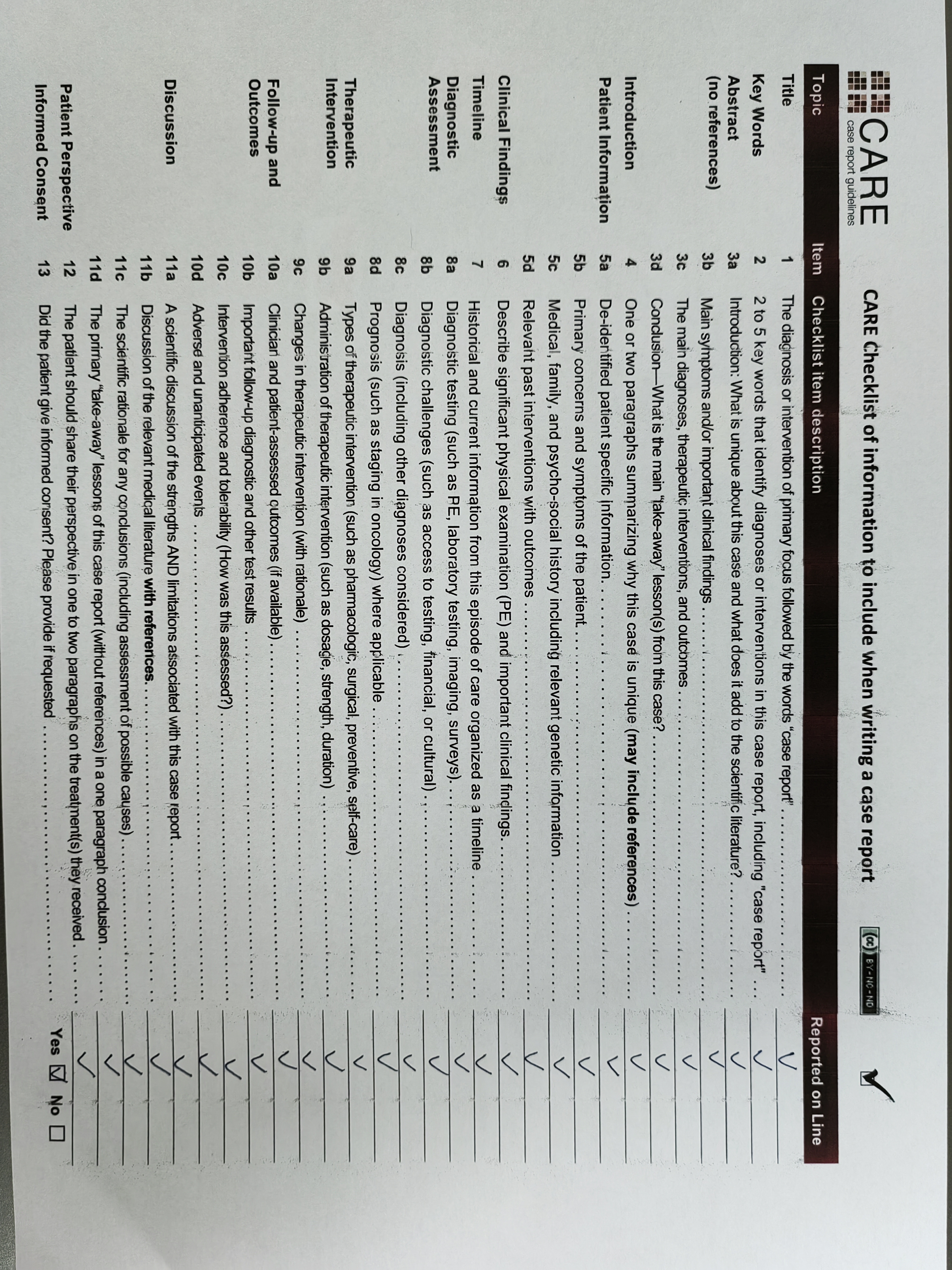

Supplement: Supplementary file 3 [file Image1.jpeg]

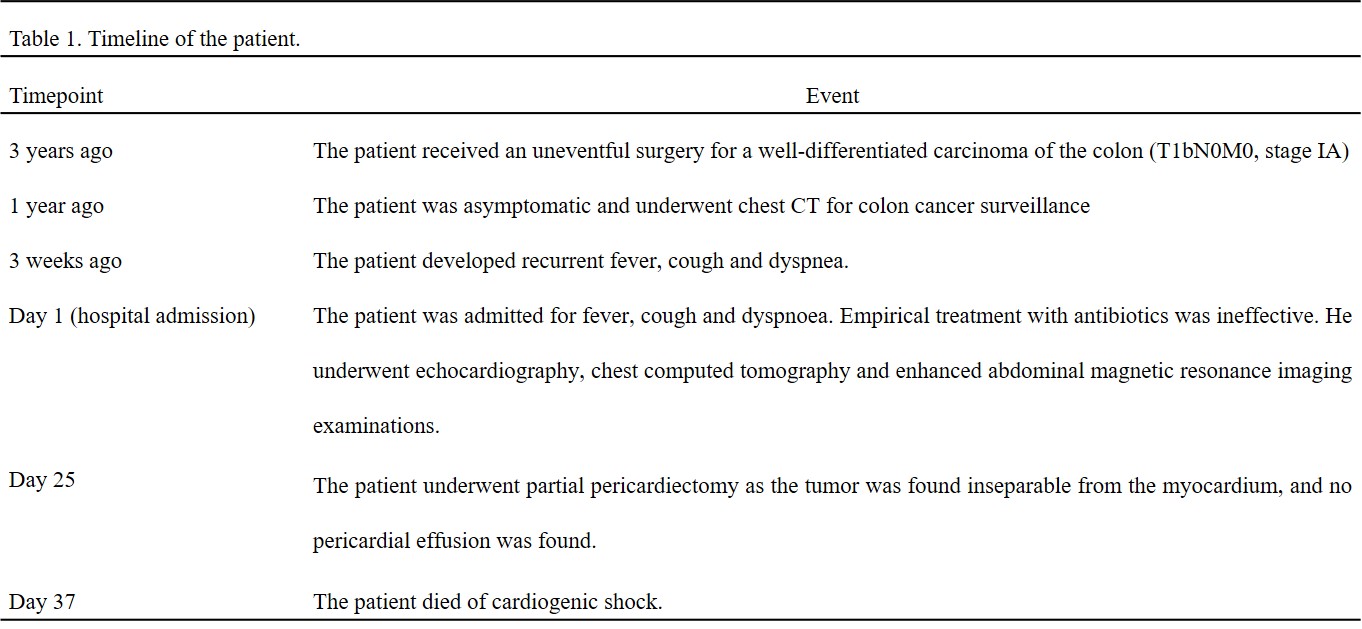

Supplement: Supplementary file 4 [file Image2.jpeg]
